# Supplementary material for: Acceptability of Prednisolone in an Open-Label Randomised Cross-Over Study—Focus on Formulation in Children
Source: Children (Basel). 2022 Aug 16;9(8):1236. doi: 10.3390/children9081236 (PMC9406412; doi:10.3390/children9081236)
Supplement: Supplementary file 1 [file children-09-01236-s001.zip › children-1839365-supplementary.pdf]

Supplemental Table S1. Other studies

Study	Population	Medicines	Findings
**Hames et al [12]**	N= 39<br>5-12 years old with asthma exacerbation<br>Unknown design, abstract only	Liquid dexamethasone 1 mg/ml<br>Liquid prednisolone 1 mg/ml	VAS scale, both medicines was rated.<br>Median VAS for dexamethasone was 8.2 cm<br>Median VAS for prednisolone was 5.0 cm (p=0.03)
**Kim et al [13]**	N = 188 for vomiting<br>N = 18/19 for taste scores<br>2-10 years old with acute asthma<br>Randomised, double blind	Generic prednisolone 15 mg/5 ml<br>Orapred 15 mg/ml	Five point face scale<br>Primary: Vomiting 17.7% generic vs 5.4% in Orapred group (RR 3.26)<br>Secondary: Median taste score for generic was 2 and for Orapred 4 (p=0.0001)
**Aljebab et al [11]**	UK N = 133 (2-16 yr)<br>Saudi Arabia (N = 122 (2-10 yr)<br>Asthma or croup, five day treatment	Either prednisolone tablets (N= 52+38), prednisolone syrup (N=37) or dexamethasone elixir (N=33) or prednisolone soluble tablets (N=42) or dexamethasone solution (N= 53)	Five point face scale<br>Dexamethasone elixir most palatable<br>Prednisolone tablets least palatable
**Lucas-Bouwmann et al [10]**	N=78<br>3 mo – 8 years with acute asthma<br>Randomised	Prednisolone solution<br>or<br>Prednisolone crushed tablets	VAS scale, oral solution Mean 3.8±0.5 (SEM) versus 7.4±0.5 (SEM) (p < 0.001).<br>Vomiting crushed tablet 23% vs 0%
